# The *Pseudomonas aeruginosa* Type III Secretion System Has an Exotoxin S/T/Y Independent Pathogenic Role during Acute Lung Infection

**DOI:** 10.1371/journal.pone.0041547

**Published:** 2012-07-23

**Authors:** Marlies Galle, Shouguang Jin, Pieter Bogaert, Mira Haegman, Peter Vandenabeele, Rudi Beyaert

**Affiliations:** 1 Department of Biomedical Molecular Biology, Ghent University, Ghent, Belgium; 2 Unit of Molecular Signal Transduction in Inflammation, Department for Molecular Biomedical Research, VIB, Ghent, Belgium; 3 Department of Molecular Genetics and Microbiology, University of Florida, Gainesville, Florida, United States of America; 4 Unit of Molecular Signaling and Cell Death, Department for Molecular Biomedical Research, VIB, Ghent, Belgium; 5 Department for Molecular Biomedical Research, Cell Culture and Sorting Core Facility, VIB, Ghent, Belgium; Indian Institute of Science, India

## Abstract

The type III secretion system (T3SS) is a complex nanomachine of many pathogenic Gram-negative bacteria. It forms a proteinaceous channel that is inserted into the host eukaryotic cell membrane for injection of bacterial proteins that manipulate host cell signaling. However, few studies have focused on the effector-independent functions of the T3SS. Using a murine model of acute lung infection with *Pseudomonas aeruginosa*, an important human opportunistic pathogen, we compared the pathogenicity of mutant bacteria that lack all of the known effector toxins ( ΔSTY), with mutant bacteria that also lack the major translocator protein PopB (ΔSTY/ΔPopB) and so cannot form a functional T3SS channel in the host cell membrane. Mortality was higher among mice challenged with ΔSTY compared to mice challenged with ΔSTY/ΔPopB mutant bacteria. In addition, mice infected with ΔSTY showed decreased bacterial clearance from the lungs compared to those infected with ΔSTY/ΔPopB. Infection was in both cases associated with substantial killing of lung infiltrating macrophages. However, macrophages from ΔSTY-infected mice died by pro-inflammatory necrosis characterized by membrane permeabilization and caspase-1 mediated IL-1β production, whereas macrophages from ΔSTY/ΔPopB infected mice died by apoptosis, which is characterized by annexin V positive staining of the cell membrane and caspase-3 activation. This was confirmed in macrophages infected *in vitro.* These results demonstrate a T3SS effector toxin independent role for the T3SS, in particular the T3SS translocator protein PopB, in the pathogenicity of *P. aeruginosa* during acute lung infection.

## Introduction

*Pseudomonas aeruginosa* is a Gram-negative bacterium found ubiquitously in soil and water habitats. It is an opportunistic pathogen that causes serious, often antibiotic resistant, infections in immunocompromised individuals, burn victims and patients requiring mechanical ventilation [1], [2]. For example, once *P. aeruginosa* is established in the airways of cystic fibrosis patients, it is almost impossible to eradicate, and the result is frequently mortality [3]. Most clinical isolates of *P. aeruginosa* secrete virulence determinants and also possess a specialized proteinaceous apparatus associated with the cell wall which is used to translocate toxins into eukaryotic cells. This is known as the type III secretion system (T3SS). The T3SS is a potent virulence mechanism shared by *Pseudomonas* and many other pathogenic Gram-negative bacteria that inject T3SS effector proteins into the cytosol of their host cells [4], [5]. The T3SS is a complex syringe-like apparatus on the bacterial surface and consists of five groups of proteins: the needle complex, the translocation apparatus, regulator proteins, chaperones and effector toxins. The needle complex is responsible for the transport of effector toxins from the bacterial cytosol to the outside. The translocation apparatus is a membrane pore that accepts the effector proteins secreted by the needle complex and delivers them across the host cell plasma membrane. The T3SS of *P. aeruginosa* uses three proteins for translocation: PopB, PopD and PcrV [6]. The latter is located at the distal end of the needle and serves as a molecular platform where PopB and PopD form the translocation pore by oligomerisation. The exact regulation of the polymerization is poorly understood. PopB, PopD and PcrV are secreted via the T3SS and are absolutely required for pore formation and translocation of effectors across the host cell plasma membrane [7], [8]. In *Yersinia* YopB, it was demonstrated that secreted translocators cannot cross-complement a *yopB* null mutant, which suggests that pore formation requires that the secreted translocators remain in close proximity to the needle [9]. The steps of triggering effector secretion upon cell contact have not been elucidated, but several events are known to occur. First, the bacterium makes contact with the cell, a process mediated by specific adhesins [9]. Then, the T3SS is brought close to the plasma membrane and the translocator proteins PopB and PopD are inserted into the host membrane to form the translocation pore [10], [11]. The needle tip protein PcrV is required for appropriate assembly and insertion of PopB and PopD into host membranes [8], [12]. After formation of the translocation pore and docking of the needle to the pore, effector secretion is triggered. Transcription and secretion of the T3SS effector proteins are regulated by specific regulator proteins. *In vitro*, secretion can be triggered by calcium depletion or by contact with host cells [13]. Proteins destined for secretion by T3SS are bound by chaperones that facilitate their storage in the cytosol and delivery to the secretion apparatus. *P. aeruginosa* has four known effector toxins: ExoS, ExoT, ExoY and ExoU. These proteins can modify signal transduction pathways and counteract innate immunity [14]. ExoS and ExoT are bifunctional enzymes with GTPase activating protein (GAP) activity and ADP ribosyl transferase (ADPRT) activity, which target several proteins, including Ras and Ras-like GTPases. These two distinct enzymatic activities work redundantly to disrupt the actin cytoskeleton, resulting in profound effects on host cellular processes [15]. While the ADPRT domains of ExoS and ExoT are highly homologous and both require the 14-3-3 family protein FAS as a cofactor, their targets are very different. In contrast to ExoS, which has poly-substrate specificity, ExoT ADP-ribosylates a more restricted subset of host proteins, including the Crk adaptor proteins. Expression of the ADPRT domain of ExoS is toxic to cultured cells, while expression of ExoT appears to interfere with host cell phagocytic activity [15]. We previously reported that ExoS negatively regulates the *P. aeruginosa* induced interleukin-1β (IL-1β) maturation and secretion by a mechanism that is dependent on its ADPRT activity [16]. ExoY is an adenylate cyclase that requires an unidentified host cell cofactor for it activity. Its role in virulence remains uncertain, though it can cause cell rounding upon cocultivation with cells [17] and is toxic when expressed in yeast [18]. ExoU has been characterized as a member of the phospholipase family of enzymes and has at least phospholipase A2 activity [19]. Similar to ExoS, ExoT and ExoY, ExoU requires either a eukaryote-specific cofactor for its activity and ubiquitinated proteins, as well as ubiquitin itself, have been suggested as being potential activators of the toxin [20]. In mammalian cells, the direct injection of ExoU causes irreversible damage to cellular membranes and rapid necrotic death. ExoS and ExoU are rarely found together in one strain. Both genotypes (*ExoS/ExoT* and *ExoU/ExoT*) are associated with acute infections in humans, though ExoU-producing strains are under-represented in persistently infected cystic fibrosis patients [21].

While much of the T3SS mediated damage is probably caused by the translocated effectors, there is increasing evidence that insertion of the needle complex itself can contribute to virulence. For example, the *Yersinia* T3SS translocator proteins YopB and YopD can form pores that allow ion influx [22] and trigger the maturation and release of pro-inflammatory IL-1β [23]. Bacterial flagellin and the inner rod component of the T3SS apparatus from several bacterial species (e.g. PrgJ from *S. typhimurium* or PscI from *P. aeruginosa*) can be detected in the host cell cytoplasm by members of a small subfamily of NLR (nucleotide-binding domain leucine-rich repeat containing) proteins, the NAIPs (NLR family, apoptosis inhibitory proteins) [24], [25]. Once activated, NAIPs appear to be critical for inducing assembly of the NLRC4 (formerly called IPAF) inflammasome, a large cytoplasmic complex that induces caspase-1 activation dependent proIL-1β maturation and pyroptotic cell death [26]. It should be mentioned that these studies were obtained with mouse cells, which express multiple NAIP paralogues. However, humans express only one NAIP and this protein was shown to recognize neither flagellin nor the inner rod (PrgJ-like) proteins of T3SS. Instead, it appears that human NAIP can detect the needle subunit of several different T3SSs, including PscF from *P. aeruginosa* and PrgI from *S. typhimurium* [24]. The current model is that different components of the T3SS are directly recognized by NAIPs but a major caveat is that evidence is mainly based on overexpression of the corresponding proteins in heterologous cell types.

Consistent with a role for the *P. aeruginosa* T3SS in caspase-1 activation, we previously showed that ΔS/ΔPopB *P. aeruginosa* (mutated in the effector protein ExoS and the translocator protein PopB), in contrast to ΔS bacteria (mutated only in ExoS), cannot activate proIL-1β maturation in a macrophage cell line [16]. However, as absence of PopB prevents the formation of a functional translocation channel as well as injection in the host cytoplasm of T3SS effector proteins other than ExoS, the exact contribution of the T3SS in the activation of caspase-1 and proIL-1β maturation is in this case unclear. Also, so far the role of the T3SS translocation pore in *P. aeruginosa* lung infection has not been studied *in vivo*. Using a murine model of acute lung infection as well as *in vitro* infection of macrophages, and comparing a mutant *P. aeruginosa* PAK strain that lacks any of the known effector toxins (ΔSTY), with mutant bacteria that also lack the major translocator protein PopB ( ΔSTY/ΔPopB), we provide evidence that T3SS plays a role in the pathogenicity of *P. aeruginosa* that is independent of the known effector toxins.

## Results

### The T3SS Translocation Pore Contributes to Mortality in Acute Lung Infection Independently of the Injection of T3SS Effector Proteins ExoS, -T, and -Y

**Figure 1:**
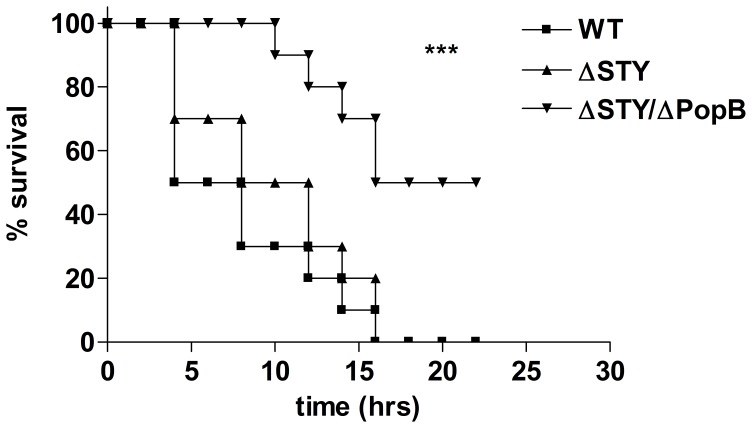
Survival of mice infected with different *P. aeruginosa* T3SS mutant bacteria. A lethal dose of 8.1^7^ cfu of WT *P. aeruginosa* or ΔSTY or ΔSTY/ΔPopB mutant strains was instillated intratracheally in C57BL/6 mice. (n = 10 for each group, NI  =  non-infected). Mortality was monitored for 24 h. Results are representative of three independent experiments. ***P≤0.001.

To investigate the toxin-independent function of the T3SS in the pathogenicity of *P. aeruginosa,* we compared the survival rates of 8-week-old C57BL/6 mice after intratracheal infection with 8.1×10^7^ cfu of a *P. aeruginosa* mutant PAK strain that lacks all known T3SS effector proteins but can still form a T3SS translocation pore (ΔSTY) and mice infected with a mutant strain that also lacks the major T3SS translocator protein PopB (ΔSTY/ΔPopB) and so cannot form a functional translocation channel. Non-infected (NI) mice instilled with PBS and mice infected with wild type (WT) bacteria were used as controls. Mortality was recorded for 24 h, after which no further deaths occurred. All NI mice survived and recovered fully from PBS instillation after 1 h. All mice challenged with WT or ΔSTY bacteria died within 16 h of challenge. ΔSTY bacteria do not produce the effector toxins ExoS, ExoT and ExoY expressed by WT bacteria, and so these T3SS effector proteins probably do not play a role in death from acute lung infection with *P. aeruginosa*. In contrast, the ΔSTY/ΔPopB mutant, which cannot form a functional T3SS translocation pore, resulted in significantly less mortality than the WT and ΔSTY strains (p = 0.0001 and p = 0.0004, respectively; Figure 1). A ΔPopB mutant behaved completely similar as the ΔSTY/ΔPopB mutant (data not shown), which is consistent with its inability to inject the T3SS effector proteins due to the lack of a functional T3SS translocation pore. These results show an important role for PopB in *P. aeruginosa* pathogenicity that is independent from its function in the secretion of ExoS, ExoT and ExoY.

### The T3SS Translocation Pore Prevents Bacterial Clearance from the Lungs Independently from the Injection of T3SS Effector Proteins ExoS, -T, and -Y

**Figure 2:**
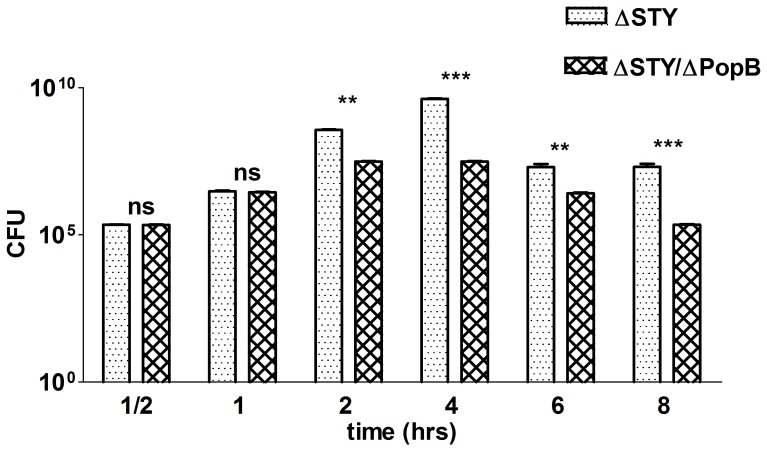
Bacterial load in the lungs of mice infected with different *P. aeruginosa* T3SS mutants. C57BL/6 mice were infected intratracheally with 1.1^6^ cfu of *P. aeruginosa* of the ΔSTY or ΔSTY/ΔPopB mutant strain. Bacterial load was determined by counting at different time points post infection (PI) the number of viable bacteria in lung homogenates. Lung homogenates were diluted serially with sterile water and placed on LB agar plates for 24 h at 37°C (n = 6 mice per group; NI  =  non-infected). Bars show means and standard deviations (SD) and are representative of 3 independent experiments. **0.001≤P≤0.01; ***P≤0.001; ns  =  not significant.

To further investigate the toxin-independent role of PopB in the virulence of *P. aeruginosa in vivo*, we compared the bacterial load in lungs of C57BL/6 mice challenged intratracheally with 1.1×10^6^ cfu of the *P. aeruginosa* ΔSTY or ΔSTY/ΔPopB strain at different times post infection. For up to 1 h after infection, the bacterial load was similarly high for both mutant strains, but later on the number of bacteria in the lungs was significantly lower in mice infected with ΔSTY/ΔPopB than in mice infected with ΔSTY (Figure 2). Bacterial titers in the blood were low at the time points measured, and no significant differences could be seen between mice infected with ΔSTY/ΔPopB or with ΔSTY (data not shown). The lower bacterial load in the lungs of mice infected with ΔSTY/ΔPopB compared to ΔSTY indicates that a functional T3SS translocation channel can prevent bacterial clearance even in the absence of any of the known T3SS effector proteins.

### The T3SS Translocation Pore Determines the Type of Pulmonary Macrophage Cell Death Independently of the Injection of T3SS Effector Proteins ExoS, -T, and -Y

**Figure 3:**
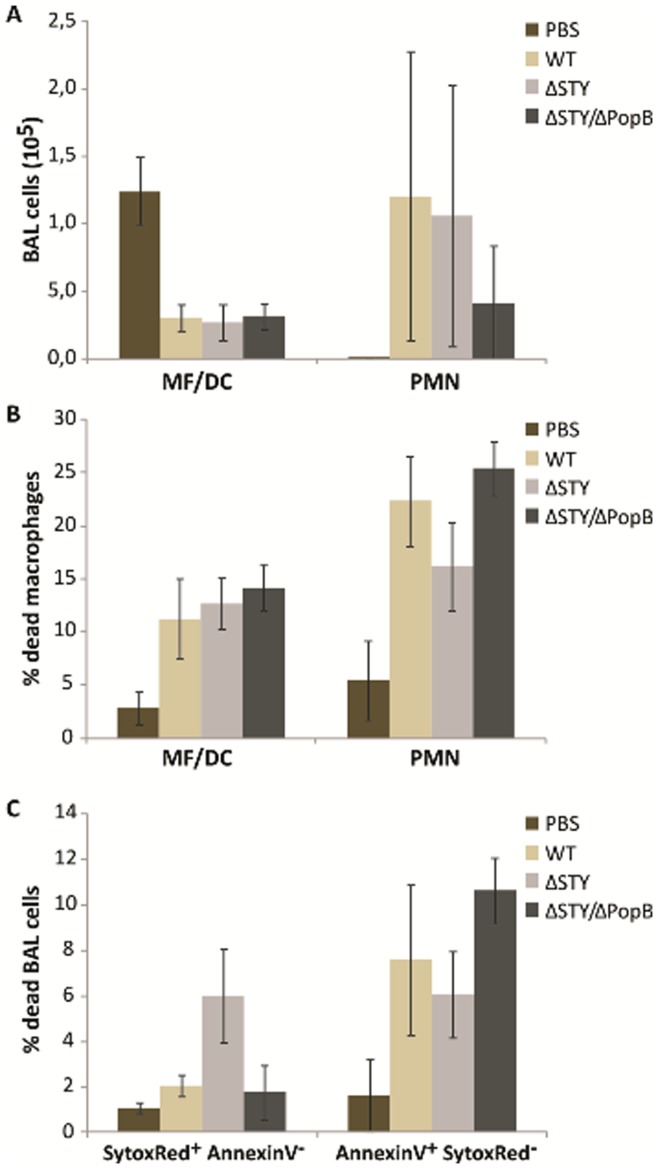
Inflammatory cell infiltration and cell death in lungs of mice infected with different *P. aeruginosa* T3SS mutants. C57BL/6 mice were infected intratracheally with 1.1^6^ cfu of *P. aeruginosa* ΔSTY or ΔSTY/ΔPopB strain for 4.5 h. The cells were analyzed by flow cytometry as described in materials and methods. **A.** The number of macrophages and PMNs in BALF was counted. **B.** Total numbers of dead macrophages and PMNs were calculated as the sum of check Annexin-V^pos^ Sytox^neg^, Annexin-V^neg^ Sytox^pos^, and Annexin-V^pos^ Sytox^pos^ cells. **C.** The mode of macrophage death was determined by separating Annexin-V^pos^ Sytox^neg^ from Annexin-V^neg^ Sytox^pos^ cells (n = 6 mice per group; NI were used as a control). The data are expressed as means (+/− SD) and are representative of 3 independent experiments.

To further investigate the observed difference in pathogenicity between ΔSTY/ΔPopB and ΔSTY in acute lung infection, we counted inflammatory cells in the BALF of C57BL/6 mice that were challenged intratracheally with 1.1×10^6^ cfu of a *P. aeruginosa* WT, ΔSTY or ΔSTY/ΔPopB strain, and after 4.5 h, neutrophils (Gr1^+^/CD11c^−^) and macrophages/dendritic cells (CD11c^+^) in BALF were counted by flow cytometry. As expected, *P. aeruginosa* infection resulted in the infiltration mainly of neutrophils. However, there were no differences between ΔSTY/ΔPopB or ΔSTY infected mice (Figure 3A). We next evaluated the viability of the inflammatory cells by labeling them with Sytox Red and Annexin-V-FITC. The Sytox Red dye only stains necrotic cells [27], whereas Annexine-V positivity indicates apoptosis [28]. *P. aeruginosa* infection resulted in significant cell death of residual macrophages and infiltrated neutrophils (Figure 3B). Overall death of macrophages and neutrophils (Annexin-V positive plus Sytox Red positive) was not significantly different between mice infected with WT, ΔSTY/ΔPopB or ΔSTY bacteria. However, the Sytox Red positive macrophage population was larger in ΔSTY infected mice than in mice infected with WT or ΔSTY/ΔPopB bacteria (p<0.01), which indicates that ΔSTY infection preferentially triggers a necrotic cell death in macrophages (Figure 3C, left panel). In contrast, the Annexin-V signal positive macrophage population was significantly larger in ΔSTY/ΔPopB infected mice than in mice infected with WT or ΔSTY bacteria (p<0.05), which indicates that ΔSTY/ΔPopB infection preferentially triggers apoptosis in macrophages (Figure 3C, right panel). There were no significant differences in the neutrophil mode of cell death after infection with WT or mutant *P. aeruginosa* strains (data not shown). All together, these data indicate that macrophage cell death can be induced by *P. aeruginosa* that lack a functional T3SS translocation channel, but implicate a role for the latter, independent of ExoS, -T and -Y, in directing cell death from apoptosis towards necrosis.

### The T3SS Translocation Pore Initiates the Maturation and Secretion of IL-1β Independent from the Injection of T3SS Effector Proteins ExoS, -T, and -Y

**Figure 4:**
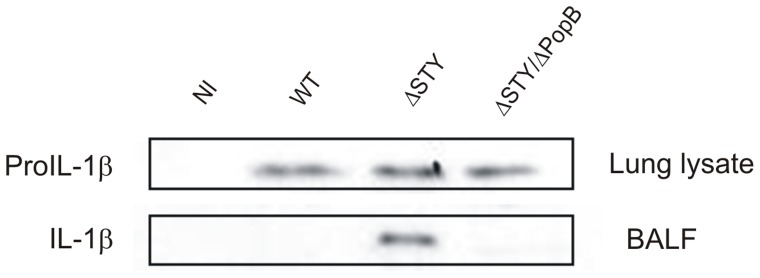
IL-1β maturation and secretion in lungs of mice infected with different *P. aeruginosa* T3SS mutants. C57BL/6 mice were infected intratracheally with 1.1^6^ cfu of WT *P. aeruginosa* or ΔSTY or ΔSTY/ΔPopB mutants, and after 4.5 h, BALF was isolated and analyzed by SDS-PAGE and western blotting for the presence of mature IL-1β. The corresponding total lung extracts were also analyzed by western blotting for the presence of pro IL-1β. Results are representative of six different mice per experimental condition.

We previously showed that the T3SS effector protein ExoS inhibits the caspase-1 mediated proteolytic maturation and secretion of IL-1β in response to *P. aeruginosa* infection [16]. Moreover, we showed that infection of a macrophage cell line with a *P. aeruginosa* a ΔPopB or ΔS/ΔPopB mutant failed to induce IL-1β maturation, which points to a role for PopB in this process. To determine whether PopB could trigger IL-1β maturation *in vivo* independently of any of the other known T3SS effector proteins, we intratracheally infected C57BL/6 mice with 1.1×10^6^ cfu of WT, ΔSTY or ΔSTY/ΔPopB *P. aeruginosa* and determined the presence of proIL-1β in total lung extracts and mature IL-1β in BALF 4.5 h post infection. Infection with all strains led to up-regulation of proIL-1β (Figure 4). However, mature IL-1β was only produced in response to infection with ΔSTY bacteria, which is consistent with our previous finding that ExoS interferes with caspase-1 mediated maturation of IL-1β [16]. Additional deletion of PopB, however, made ΔSTY bacteria unable to induce IL-1β maturation. These data indicate that the formation of a functional T3SS translocation channel triggers the activation of IL-1β maturation, independently from any of the known T3SS effector proteins.

### The T3SS Translocation Pore Affects Cell Death and IL-1β Maturation of Cultured Macrophages, Independent of the Injection of T3SS Effector Proteins ExoS, -T, and –Y

**Figure 5:**
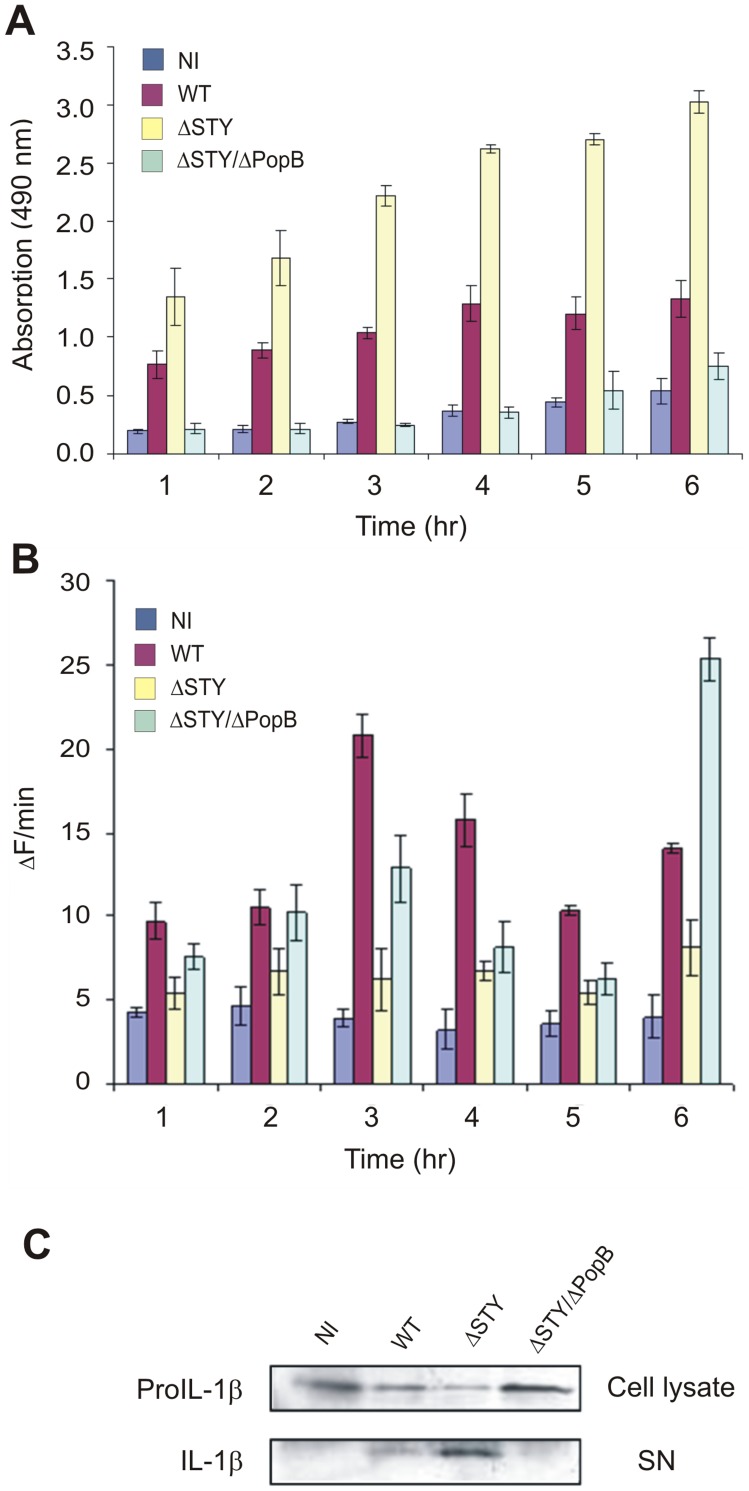
Cell death and IL-1β maturation upon infection of the MF4/4 macrophage cell line with different *P. aeruginosa* T3SS mutants. MF4/4 cells were pre-stimulated with 100 ng/ml LPS for 4 h and infected with WT *P. aeruginosa* or ΔSTY or ΔSTY/ΔPopB mutant strains at a MOI of 100. Non infected cells prestimulated with 100 ng/ml LPS for 4 h were used as a control. **A.** LDH release in the culture medium was determined by spectrophotometry as described in materials and methods and is expressed as optical density (absorbance) at 490 nm. Results are the mean +/− SD of triplicates and are representative for four independent experiments. **B.** Caspase-3 activity in cell extracts was measured in a fluorometric assay with Ac-DEVD-AMC and is expressed as change in fluorescence over time (Δf/min). Results are the mean +/− SD of triplicates and are representative of three independent experiments. **C.** Culture supernatants (SN) were collected and analyzed for the presence of mature IL-1β by immunoprecipitation of IL-1β followed by SDS-PAGE and western blotting. The corresponding total cell lysates were analyzed for the presence of proIL-1β by western blotting. Results are representative of three independent experiments.

To further explore whether the above described effect of PopB in the murine model of *P. aeruginosa* lung infection reflects a direct effect on macrophages, we compared the same ΔSTY/ΔPopB and ΔSTY mutant strains for their ability to cause macrophage death and IL-1β maturation *in vitro*. The macrophage MF4/4 cell line was infected with *P. aeruginosa* at a multiplicity of infection (MOI) of 100. Necrosis-like cell death, associated with membrane permeabilization, was measured by LDH release in the medium. Apoptosis, associated with caspase-3 activation, was measured by determining caspase-3 activity in total cell lysates using the fluorescent peptide substrate Ac-DEVD-AMC. Cell death was followed for 6 h after infection, at which time about half of the ΔSTY infected cells were floating in the medium. WT and ΔSTY infected cells showed substantial LDH release, which was more pronounced in the case of ΔSTY infection (Figure 5A). However, ΔSTY/ΔPopB infected cells did not show any LDH release. In contrast, there was biphasic activation of caspase-3 in WT and ΔSTY/ΔPopB infections, but not in ΔSTY infection (Figure 5B). Finally, we also tested the proteolytic maturation of proIL-1β in these cells. Because the strong cytotoxic effect prevented the induction of proIL-1β, we pretreated the cells with 100 ng/ml LPS for 4 h, which induced the expression of proIL-1β but not its maturation. WT bacteria induced only marginal maturation of IL-1β, which was significantly higher in ΔSTY infected cells. In contrast, additional deletion of PopB (ΔSTY/ΔPopB) inhibited the ability from the pathogen to induce IL-1β maturation (Figure 5C). All together, these *in vitro* results are consistent with the results obtained in the *in vivo* lung infection model, which indicates that the T3SS translocation channel has a direct function that is independent of the known T3SS effector proteins in *P. aeruginosa* infected macrophages.

## Discussion

Much effort has been put in the functional and structural characterization of the T3SS of *P. aeruginosa* and other Gram-negative bacteria, and its interaction with the host cell. These studies have focused mainly on the role of the T3SS effector proteins (ExoS, ExoT, ExoY, ExoU in the case of *P. aeruginosa*) in virulence. While this manuscript was under revision, T3SS rod and needle proteins were shown to trigger the activation of the NAIP/NLRC4 inflammasome when expressed in mammalian cells [24]–[26], illustrating functions of the T3SS beyond the injection of T3SS effector proteins. In the present study, we compared the pathogenicity of *P. aeruginosa* WT bacteria (PAK strain, which is deficient for ExoU) with that of mutant bacteria ΔSTY (devoid of ExoS, ExoT and ExoY) and ΔSTY/ΔPopB (devoid of ExoS, ExoT, ExoY, and the T3SS translocator protein PopB). The ΔSTY/ΔPopB mutant has an intact needle complex but cannot form pores in host cells or translocate T3SS effector proteins. Using a murine model of acute lung infection as well as infection of cultured macrophages with the above described mutants, we provide evidence that the T3SS translocation pore plays an important role in *P. aeruginosa* pathogenicity that is independent of the injection of any of the known T3SS effector proteins. This is illustrated by the findings that compared to ΔSTY/ΔPopB, infection with ΔSTY leads to higher mortality, reduced bacterial clearance, and non-apoptotic killing of alveolar macrophages, which is associated with the production of pro-inflammatory IL-1β. Absence of IL-1β production in ΔSTY/ΔPopB infected mice might contribute to the more efficient clearance of the bacteria from the lungs and the lower mortality compared to ΔSTY infected mice. This is consistent with previous data showing that absence or reduction of endogenous IL-1β activity improves host defense against *Pseudomonas* pneumonia while suppressing the inflammatory response [29]. The extended life of ΔSTY/ΔPopB infected mice compared to ΔSTY infected mice may therefore not only reflect a reduction in the bacterial burden but also the observed reduction in the amount of IL-1β, which is known to trigger several signaling cascades leading to massive inflammation and multiple organ failure. In this context, it was previously shown that several cytokines, among them IL-1β, were present at significantly higher levels in BALF from mice infected with bacteria possessing an intact T3SS than in mice infected with a *PcrV* mutant that is unable to form a translocation pore [30]. We previously showed that ExoS interferes with the *P. aeruginosa* induced activation of caspase-1 and the production of IL-1β in a macrophage cell line [16]. However, we did not observe a significant difference in survival between WT-infected and ΔSTY-infected mice in the current study. It is likely that the relative importance of ExoS in immune evasion depends on specific factors such as bacterial load and site of infection, or differs in acute versus chronic infection. The specific role of ExoS in immune evasion awaits further studies directly comparing infection under different conditions.

Consistent with the lower mortality seen in mice infected with ΔSTY/ΔPopB compared to those infected with ΔSTY, the ΔSTY/ΔPopB bacteria were more efficiently cleared from the lungs. Bacterial clearance is mediated mainly by neutrophils and macrophages. *P. aeruginosa* lung infection has been shown to induce the recruitment of neutrophils [31], which we confirmed in our study. Abolishing neutrophil recruitment, due to the T3SS, decreases bacterial clearance [30], [32]. However, we did not find a significant difference in the number of alveolar neutrophils between mouse infections with WT, ΔSTY or ΔSTY/ΔPopB. It should be noted that the previously observed T3SS-induced decrease in neutrophils occurred 12 h after infection, which is much later than the time (4 hours) at which we observed a difference in bacterial clearance. Several studies reported an important function of alveolar macrophages in *P. aeruginosa* induced pneumonia [31], [33], [34]. Here, we show that the number of residual alveolar macrophages is significantly decreased in *P. aeruginosa* infected mice, consistent with increased macrophage cell death. A contribution of the T3SS to the killing of macrophages has been described in animal models of *P. aeruginosa* acute lung infections [35] and in clinical studies of lower respiratory tract infections and ventilator-acquired pneumonia [36], [37]. However, these studies attributed the role of the T3SS to the activity of T3SS effector proteins that are injected in the host cell cytosol, and did not study a potential T3SS effector independent function of the T3SS. Our data show that the formation of the T3SS translocation pore itself, rather than the injection of T3SS effector proteins, is the main trigger of macrophage cell death. Interestingly, although the total number of dying alveolar macrophages was comparable in WT, ΔSTY and ΔSTY/ΔPopB infected mice, there were significant qualitative differences. In the absence of T3SS effector proteins, a significantly larger fraction of the cells showed necrotic rather than apoptotic features In addition, necrosis-like alveolar macrophage cell death was also associated with caspase-1 mediated maturation and secretion of the pro-inflammatory cytokine IL-1β, which is generally described as pyroptosis [38]. These findings are consistent with our previous *in vitro* observation of infection of cultured macrophages with ExoS deficient bacteria switching the mode of cell death from apoptosis to necrosis [16]. However, the polarization towards necrosis was again lost upon additional deletion of PopB. The above described differences in cell death and IL-1β production were confirmed *in vitro* in a murine macrophage cell line, suggesting that the *in vivo* differences reflect a direct effect of the bacteria on macrophages. Together, these data indicate that *P. aeruginosa* can trigger apoptosis independently of the formation of a T3SS translocation pore and the injection of T3SS effector proteins. In addition, PopB-mediated formation of a T3SS translocation pore, without injection of the T3SS effector proteins ExoS, ExoT or ExoY, induces pro-inflammatory necrosis. However, as soon as T3SS effector proteins are injected, necrosis is rapidly switched to non-inflammatory apoptosis, most likely as a result of ExoS-mediated inhibition of caspase-1. The T3SS-independent apoptosis observed upon infection with ΔSTY/ΔPopB bacteria might be triggered by other toxins that are secreted by *P. aeruginosa*, such as pyocyanin, which induces apoptosis of neutrophils [39], [40]. Necrosis initiated by ΔSTY bacteria excludes a role for any of the four known T3SS effector proteins. Despite extensive characterization of the *P. aeruginosa* T3SS in several laboratories, no other T3SS effector proteins have been identified. Cell membrane permeabilization by the translocation pore has also been suggested to trigger ion fluxes or other events [8], [41]–[43], some of which might signal necrosis. Alternatively, we cannot exclude the possibility that PopB has a direct effect that is independent from the formation of the pore. In this context, IL-8 production induced by *Yersinia pseudotuberculosis* has been shown to require YopB, a functional homolog of PopB, while pore formation is not required [44]. Furthermore, binding of caspase-1 with the PopB homologs SipB and IpaB has been suggested to contribute to caspase-1 activation in response to *Salmonella* and *Shigella* infection [45], [46], respectively. However, by co-immunoprecipitation we were unable to demonstrate binding between caspase-1 and PopB (data not shown). Recently, T3SS rod and needle proteins were found to be sensed in the host cell cytoplasm by specific members of the NAIP protein family, which then triggers activation of the NLRC4 inflammasome that is responsible for caspase-1 mediated proIL-1β maturation and pyroptotic cell death [24]–[26]. It is therefore tempting to speculate that also the T3SS translocator protein PopB is similarly sensed by a specific NAIP protein, leading to inflammasome activation.

Resistance to antibiotics is a major problem in the treatment of *P. aeruginosa* infections in critically ill patients. It might be possible to develop T3SS inhibitors as novel antibacterial therapeutics with modes of action distinct from conventional antibiotics. Administration of monoclonal antibodies against the T3SS needle-tip protein PcrV has previously demonstrated efficacy against laboratory strains of *P. aeruginosa* in a murine model [47], and recently it entered Phase I/II clinical trials [48]. Efforts have been directed at developing inhibitors that block other aspects of the *P. aeruginosa* T3SS. For example, small molecule inhibitors of ExoU’s phospholipase activity and ExoS’s ADPRT activity have been identified, and each one could protect against killing of the host in models of *P. aeruginosa* infection [18], [49]. Our results show that targeting the T3SS effector proteins might not be the best approach and indicate that interfering with the formation or activity of the T3SS translocation pore might be a better approach. Recently, screening of a compound library resulted in the discovery of five compounds that inhibit the activity of the *P. aeruginosa* T3SS apparatus and the secretion of all its effector proteins [50]. The molecular targets of these inhibitors are not known, but the observed spectrum of activity against T3SS in three bacterial species points to a conserved target, possibly PopB.

## Methods

### Ethics Statement

All experiments were approved by and performed in accordance with the guidelines of the animal ethical committee of Ghent University (permit number LA1400091, approval ID 04/12). All efforts were made to ameliorate suffering of animals. Mice were anesthetized by intraperitoneal injection of a mixture of ketamine (100 mg/kg) and xylazine (10 mg/kg).

### Bacterial Strains

The *P. aeruginosa* laboratory strain PAK (which has the effector toxins ExoS, ExoT, ExoY but lacks the *ExoU* gene) and mutants thereof with specific chromosomal deletions *ΔExoS/ΔExoT/ΔExoY* or *ΔExoS/ΔExoT/ΔExoY/ΔPopB* have been described [51], [52]. Bacterial cultures were grown in Luria–Bertani broth (LB) at 37°C.

### *In vitro* Infection of MF4/4 Cells

The immortalized murine macrophage cell line MF4/4 [53] was cultured at 37°C with 5% CO_2_ in RPMI 1640 supplemented with 10% fetal bovine serum, 2 mM L-glutamine, 100 U/ml penicillin, 100 µg/ml streptomycin sulfate, 1 mM sodium pyruvate and 2×10^−5^ M β-mercaptoethanol. Cells were seeded at 10^6^ cell per well in six-well plates and 4 h before infection they were stimulated with 100 ng/ml LPS. Cells were infected with *P. aeruginosa* at a multiplicity of infection of 100. Before lysing cells and collecting supernatants, bacteria were killed by adding 100 µg/ml gentamicin and 50 µg/ml chloramphenicol. Cells were lysed in 250 µl lysis buffer (50 mM Hepes pH 7.6, 200 mM NaCl, 0.1% NP40, and 5 mM ethylenediaminetetraacetic acid [EDTA]).

### Mouse Model of Acute Pneumonia

Eight-week-old female C57BL/6 mice (BioServices Janvier, Schuijk, The Netherlands) that were kept under ‘Specific Pathogen Free’ conditions were intratracheally instillated with *P. aeruginosa* as described [54]. Bacterial cultures were grown overnight in LB medium at 37°C, diluted 1/100, and grown to exponential phase. Bacteria were collected by centrifugation and re-suspended to the appropriate density of colony forming units (cfu)/ml in phosphate buffer saline (PBS), as determined by optical density measurement and plating of serial dilutions on Nutrient Broth agar plates. Mice were anesthetized by intraperitoneal injection of a mixture of ketamine (100 mg/kg) and xylazine (10 mg/kg). Bacterial solutions were administered by intratracheal instillation of 1×1^6^ cfu (for measurement of bacterial clearance) or 8×10^7^ cfu (for survival assays) in 50 µl of PBS. Survival rate was followed for 48 hours after infection. Bacterial clearance was measured by determining the number of bacteria in lung homogenates at different times post infection. Lung homogenates were diluted serially and cultured on agar plates for 24 h at 37°C for assessment of the bacterial load.

### Bronchoalveolar Lavage and Preparation of Lung Extracts

After the indicated times of infection the trachea was exposed through a midline incision and a sterile catheter was inserted. Bronchoalveolar lavage was performed by instilling two aliquots of 0.5 ml PBS. BALF was retrieved, centrifuged and filtered (syringe 0.22-µm filter, Millipore, Bedford, USA). The lungs were homogenized in 1 ml PBS and Laemmli loading buffer was added for western blot analysis.

### IL-1β Immunoprecipitation

IL-1β was immunoprecipitated from the cell supernatant by the addition of 10% lysis buffer (50 mM Hepes pH 7.6, 200 mM NaCl, 0.1% NP40, and 5 mM ethylenediaminetetraacetic acid [EDTA]) and incubation for 15 h with 2 µg of a rat monoclonal anti-IL-1β antibody (MAB401, R&D systems), followed by incubation with protein G-sepharose (Amersham Biosciences) for 3 h. The beads were washed four times with lysis buffer before elution with Laemmli buffer.

### Western Blotting

Total cell extracts, BALF and IL-1β immunoprecipitations were separated by SDS-PAGE and analyzed by western blotting and immunodetection with anti-IL-1β antibodies (AF-401-NA, R&D systems). Total cell extracts and IL-1β immunoprecipitates were separated by SDS-PAGE and analyzed by western blotting and immunodetection with anti-IL-1β antibodies (AF-401-NA, R&D systems) and HRP-linked anti-mouse Ig (Amersham Biosciences). Immunoreactivity was revealed with the enhanced chemiluminescence method (NENTM Renaissance, NEN Life Sciences Products).

### Analysis of Caspase-3 Activity and Lactate Dehydrogenase Release

Caspase-3-like activity was measured by incubating cell extracts with Ac-DEVD-AMC and monitoring the release of 7-amino-4-methyl coumarin (AMC) in a fluorometer as described previously [55].

Lactate dehydrogenase (LDH) release in the culture supernatants of cells at the indicated time points after infection was quantified by using a cytotoxicity assay kit (Cytotox 96; Promega, Charbonnieres, France).

### Flow Cytometric Analysis of Bronchoalveolar (BAL) Immune Cell Subsets and Cell Death Type

BAL macrophage/dendritic cell (MF/DC) and neutrophil (PMN) populations and the mode of cell death were analyzed by flow cytometry on a triple-laser (405 nm, 488 nm, 635 nm) LSR-II with FACSDiva software (BD Biosciences, San Jose, CA, USA). After the indicated times of infection, BALF was retrieved and concentrated by centrifugation. Cells were kept on ice and labeled with anti-CD16/CD32 to block Fc receptor binding, PE-conjugated anti-Gr1 and PerCP-Cy5.5-conjugated anti-CD11c (all BD Pharmingen) at 4°C in the dark. After washing, cells were dissolved in Annexin-V binding buffer with 0.02% MgCl_2_, 0.03% CaCl_2_, 0.9% NaCl, 0.037% KCl, 0.024% sodium azide and 0.38% NaOH-Hepes. Alexa Fluor 488-conjugated Annexin-V (5 µl) and the cell-impermeant Sytox Red Dead Cell stain (5 nM) (both Invitrogen) were then added. MF/DC were identified as cells that were CD11c^pos^, PMN as CD11c^neg^ Gr1^hi^. Viable cells were identified as Annexin-V^neg^ Sytox^neg^, early apoptotic as Annexin-V^pos^ Sytox^neg^, early necrotic as Annexin-V^neg^ Sytox^pos^, and late apoptotic/necrotic as Annexin-V^pos^ Sytox^pos^.

### Experimental Groups and Statistical Analysis

Animals were randomly assigned to the experimental groups, with a minimum of six animals per group. Differences in bacterial loads between ΔSTY and ΔSTY/ΔPopB infected mice were analyzed by the Mann-Whitney test where appropriate *P* values <0.05 were regarded as statistically significant. Cell death and cell death mode, measured on FACS, was analyzed with the Kruskall wallis test where appropriate *P* values of <0.05 were regarded as statistically significant. Survival curves (Kaplan-Meyer plots) were compared using a log-rank test. Final mortality counts were compared with a Chi square test. Means and standard deviation (SD) were compared with a student t test with 95% confidence intervals. *, **, and *** represent 0.01≤P≤0.05, 0.001≤P≤0.01, and P≤0.001, respectively.
